# Convolutional neural network based on SMILES representation of compounds for detecting chemical motif

**DOI:** 10.1186/s12859-018-2523-5

**Published:** 2018-12-31

**Authors:** Maya Hirohara, Yutaka Saito, Yuki Koda, Kengo Sato, Yasubumi Sakakibara

**Affiliations:** 1 Department of Biosciences and Informatics, Keio University, Yokohama, 223-8522 Japan; 2 Artificial Intelligence Research Center, National Institute of Advanced Industrial Science and Technology (AIST), Tokyo, 135-0064 Japan; 3 Computational Bio Big-Data Open Innovation Laboratory (CBBD-OIL), National Institute of Advanced Industrial Science and Technology (AIST), Tokyo, 169-8555 Japan

**Keywords:** Convolutional neural network, Chemical compound, Virtual screening, SMILES, TOX 21 Challenge

## Abstract

**Background:**

Previous studies have suggested deep learning to be a highly effective approach for screening lead compounds for new drugs. Several deep learning models have been developed by addressing the use of various kinds of fingerprints and graph convolution architectures. However, these methods are either advantageous or disadvantageous depending on whether they (1) can distinguish structural differences including chirality of compounds, and (2) can automatically discover effective features.

**Results:**

We developed another deep learning model for compound classification. In this method, we constructed a distributed representation of compounds based on the SMILES notation, which linearly represents a compound structure, and applied the SMILES-based representation to a convolutional neural network (CNN). The use of SMILES allows us to process all types of compounds while incorporating a broad range of structure information, and representation learning by CNN automatically acquires a low-dimensional representation of input features. In a benchmark experiment using the TOX 21 dataset, our method outperformed conventional fingerprint methods, and performed comparably against the winning model of the TOX 21 Challenge. Multivariate analysis confirmed that the chemical space consisting of the features learned by SMILES-based representation learning adequately expressed a richer feature space that enabled the accurate discrimination of compounds. Using motif detection with the learned filters, not only important known structures (motifs) such as protein-binding sites but also structures of unknown functional groups were detected.

**Conclusions:**

The source code of our SMILES-based convolutional neural network software in the deep learning framework Chainer is available at http://www.dna.bio.keio.ac.jp/smiles/, and the dataset used for performance evaluation in this work is available at the same URL.

## Background

In recent years, not only in vivo and in vitro but also in silico analysis, especially machine learning, which can predict chemical properties, has become increasingly important for chemical analysis. For example, predicting compound-protein interaction facilitates the screening of new lead compounds for drug discovery.

In the case of *in silico* analysis, several digital file formats are defined to enable computers to read chemical compounds. Among these formats, MOL, SDF, Fingerprints, and SMILES (Simplified Molecular Input Line Entry System) are the most widely used. MOL is a file format that represents a compound in the form of a graph connection table: each node represents an atom and the edges are the bonds between atoms. SDF is an extended version of MOL for writing multiple compounds into one file.

A “fingerprint” is a vector that represents a property of a chemical compound. Many methods for creating fingerprints have been reported. The launch pad we normally use for all fingerprints is 2D fingerprint to indicate what kind of partial structure the compound possesses. In this regard, the most commonly used algorithm is the extended-connectivity fingerprint (ECFP, also known as the circular fingerprint or Morgan fingerprint) [1]. This algorithm first searches the partial structures around each atom recurrently, then assigns an integer identifier to each partial structure, and writes this as a binary vector by using a hash function. Potentially, an infinite number of structures exist in the chemical space; consequently, ECFP requires vectors with a large number of bits (usually 1024–2048 bits). A more advanced version of the algorithm, 3D fingerprint, encodes 3D information, including the molecular shape and electrostatics. For example, ROCS (Rapid Overlay of Chemical Structures) uses “color” features defined by a simple force field [2]. A related method is USR (Ultrafast Shape Recognition), which calculates the 3D similarity without an alignment of chemical structures [3].

SMILES, which was proposed by Weininger [4], is currently widely recognized and used as a standard representation of compounds for modern chemical information processing. SMILES provides a linear notation method to represent chemical compounds in a unique way in the form of strings over a fixed alphabet. SMILES uses specific grammar and characters to describe all the atoms and structure of a chemical compound. SMILES can strictly express structural differences including the chirality of compounds. Such a linear structure of SMILES representation, referred to as a *SMILES string*, enables the straightforward application of convolutional neural network (CNN) to virtual screening of chemical compounds and identification of functional substructures, which we name *chemical motifs*.

Chemical analysis with machine learning continues to be actively researched and is motivated by contests such as the Merck Molecular Activity Challenge 2013 and TOX 21 Challenge 2014, at which the results obtained with deep neural networks were superior to those achieved with other architectures [5, 6]. However, these methods do not make full use of the capability of deep learning. Deep learning typified by CNN would benefit from the capability of automatically acquiring the features from data as much as possible instead of manually devising the features. This capability (known as *representation learning*) became a springboard for the development of machine-learning-based fingerprinting techniques focusing on the graph structure of compounds as an alternative to manually-designed fingerprints [7–9]. Duvenaud et al., [10] defined a way to generalize fingerprints with a backpropagation convolutional network. Kearnes et al., [11] improved fingerprints by using graph convolution. These methods are useful to aim for the goal of acquiring fingerprints by machine learning. However, they have one or more limitations: (1) some models can input only a set of compounds with fixed structure, (2) some cannot distinguish among stereoisomers, and (3) most importantly, the graph structure is in general not a data structure of grid-like topology, such as two-dimensional images (2-D grid of pixels), for which CNN could be used effectively.

**Fig. 1:**
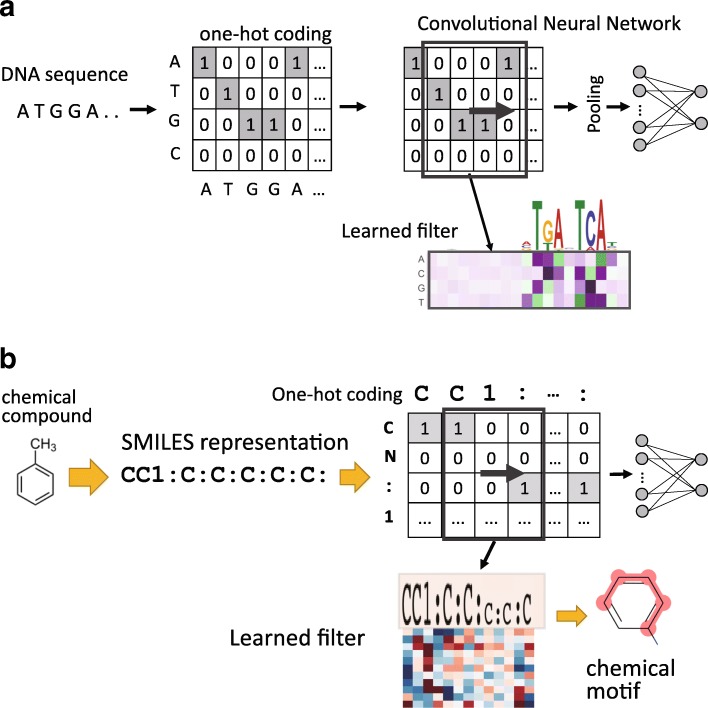
Chemical motif detection by CNN in comparison with sequence motif detection. **a** One-hot coding representation of four DNA nucleotides, a filter (kernel) with a one-dimensional convolution operation that is considered a position weight matrix for representing a motif. **b** The same strategy for applying one-dimensional CNN to SMILES linear representations of chemical compounds and the extraction of learned filters to discover the chemical motifs

The above observations led us to propose a new approach using the SMILES linear representation of chemical compounds to apply CNNs for the classifications of chemical compounds and the detection of chemical motifs. A string is the simplest grid-like (1-D grid) structure, and molecular sequences such as DNA and protein sequences are also strings. CNNs have already been applied to the classification of DNA sequences and extraction of a sequence motif conserved among the DNA sequences [12–16]. In these methods, by employing one-hot coding representation of four DNA nucleotides, a filter (kernel) with a one-dimensional convolution operation applied to a sequence can be considered a position weight matrix for representing a motif. The filters are learned by training CNNs on positive and negative samples of sequences such as those obtained in experiments on chromatin immunoprecipitation with high-throughput sequencing (ChIP-seq) [16]. Here, a “one-dimensional” convolutional operation for sequences is interpreted as scanning the input sequence only in one direction along the sequence with a filter of the same width (dimension) as that of the distributed representation of input (see Fig. 1a). Now, our approach is straightforward to simply apply one-dimensional CNN to the SMILES strings representing chemical compounds for the classification of these chemical compounds and extraction of the chemical motifs (structures) conserved among the compounds (see Fig. 1b).

We experimented with the TOX 21 dataset and evaluated the results by using the ROC-AUC score. The evaluation showed that our method, one-dimensional CNN using the SMILES representation, was superior to the ECFP fingerprint methods and graph convolution method [11]. Furthermore, several important known structures (motifs) such as protein-binding sites were detected from the learned filters in the one-dimensional CNN.

Another advanced feature of CNN is representation learning [17]. Representation learning is a procedure in which the effective features can be automatically discovered in the process of machine learning. Thus, representation learning enables us to extensively obtain new fingerprints or descriptors for compounds that fit the prediction model (e.g., prediction of binding to a certain protein). Furthermore, it is possible to extract the “chemical motif,” which is an important functional substructure (e.g., the site at which a protein could bind). We showed that the new fingerprints discovered by representation learning based on SMILES representation provided a richer chemical space that enabled the accurate discrimination of compounds, whereas existing methods using ECFP were unable to express the properties of compounds. Here, “chemical space” is a term often used in the place of “multi-dimensional descriptor space”.

## Methods

**Fig. 2:**
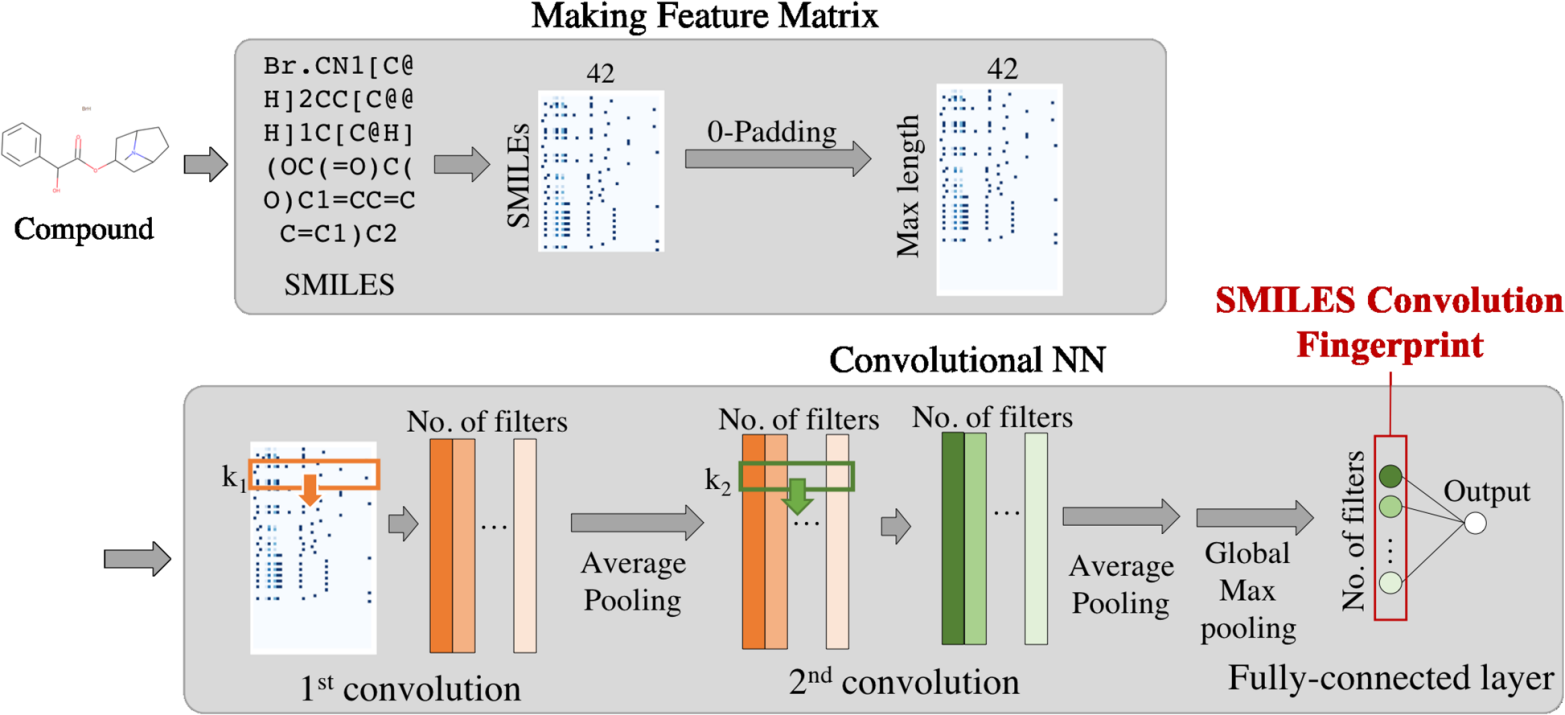
Overview of our CNN. The SMILES string of a compound is represented as a feature matrix. CNN has multiple layers consisting of two convolutional and pooling layers with a subsequent global pooling layer. CNN is applied to the feature matrix and produces a low-dimensional feature representation (actually, 64-dimensional vector) termed the SCFP. Classification models are constructed by using SCFP as input for subsequent fully connected layers

In this section, we describe a new convolutional neural network (CNN) based on the SMILES notation of compounds. An overview of our CNN is shown in Fig. 2. The main idea of our method is that we represent a SMILES string as a distributed representation termed a *SMILES feature matrix*, and apply CNN to the matrix in a way similar to the application of conventional CNNs to image data. Our CNN transforms the SMILES feature matrix into a low-dimensional feature vector termed the *SMILES convolution finger print* (SCFP). We construct classification models for compounds by using the SCFP as input for subsequent fully connected layers. In addition, we propose a novel method for extracting the acquired feature representation from our CNN as a form of “chemical motif.”

### SMILES notation for representing chemical compounds

SMILES uses two sets of symbols: a set of atomic symbols and a set of SMILES original symbols. In SMILES representation, atoms are represented by their atomic symbols, and double bonds are written using “=” and triple bonds using “#”, both of which are original SMILES symbols. Rings are represented by breaking one of the bonds in each ring, and the presence of the ring is indicated by appending an integer to each of the two atoms of the broken bond. The presence of a branch point is indicated by a left-hand bracket “(” and the right-hand bracket “)” indicates that all the atoms in that branch have been visited. We refer to a SMILES representation of a chemical compound as a *SMILES string* for the chemical compound. For example, the SMILES string for Aspirin is CC(=O)OC1=CC=CC=C1C(=O)O. Although ambiguity may occur in that a compound may be represented in more than one way using SMILES (generic SMILES), we use a normalization algorithm to ensure that one single SMILES representation is derived from one compound (this approach is also known as unique SMILES, canonical SMILES, or absolute SMILES) [18].

### SMILES feature matrix

The input used for the CNN consists of a distributed representation of a SMILES string, which comprises a sequence of feature vectors representing the symbols that occur in the SMILES string.

First, the input compound is represented by a SMILES string. Next, for each symbol in the SMILES string, a feature vector that is a distributed representation of the symbol is calculated. Each feature vector consists of 42 features, of which 21 features are used as symbols for atoms, and the remaining 21 features are used for original SMILES symbols. Each dimension in the 21-dimensional vector for an atom consists of the type of atom, and its degree, charge, and chirality, and each 21-dimensional vector for an original SMILES symbol is a one-hot vector that is a distributed representation of 21 original SMILES symbols. Note that one-hot vector is a binary vector with a single high (1) bit and all the others low (0). The 42 elements are listed in Table 1. Numerical values related to atomic substance quantities such as degree, charge, and chirality were calculated using the program RDKit (version: 2016.09.4) [19]. The length of the feature matrix is set to the maximum length of SMILES strings for compounds in a given dataset (400 in this study). In the feature matrix for SMILES strings of which the length is shorter than the maximum length, all the blank parts were padded with 0 to retain the input size. The resulting distributed representation is a two-dimensional feature matrix with the fixed size of (400, 42).

**Table 1:** Features

Feature	Description	Size
Atom		21
Atom type	H, C, O, N, or others	5
NumHs	Total number of H atoms attached to it	1
Degree	Its degree of unsaturation	1
Charge	Its formal charge	1
Valence	Its total valence	1
Ring	Whether it is included in a ring	1
Aromaticity	Whether it is included in an aromatic structure	1
Chirality	R, S, or others	3
Hybridization	*s*, *sp*, *sp*^2^, *sp*^3^, *sp*^3^*d*, *sp*^3^*d*^2^, or others	7
SMILES original symbol		21
(	Branch start	1
)	Branch end	1
[	Atom or atom group start	1
]	Atom or atom group end	1
.	Ionic bond	1
:	Aromatic bond	1
=	Double bond	1
#	Triple bond	1
\	cis	1
/	trans	1
@	Chirality (above or below)	1
+	Cation (positive ion)	1
-	Anion (negative ion)	1
Ion charge	Numbers show ionic charge (2-7)	6
Start	Numbers show ring start	1
End	Numbers show ring end	1

### CNN

Figure 2 shows the architecture of our CNN. We used multiple layers consisting of two convolutional and pooling layers with a subsequent global pooling layer. In the first convolutional layer, we used filters with the same width as that of the SMILES feature matrices (i.e., 42). This ensured that convolution was performed only for the direction of SMILES strings. In the global pooling layer, we used global max pooling [20]. Our CNN has several hyperparameters including the window size of filters, the number of filters, and others. These hyperparameters were summarized in Table 2, and determined by using Bayesian optimization, GpyOpt [21].

**Table 2:** Model hyperparameters

Hyperparameter	Considered values
1st convolution	
No. of filters	[1,1024]
Window size	[1,51]
Stride size	{1,3,5}
Padding	{None, Half of window size}
1st pooling	
Type	{Max, Average}
Window size	[1,51]
Stride size	{1,3,5}
Padding	{None, Half of window size}
2nd convolution	
No. of filters	[1,1024]
Window size	[1,51]
Stride size	{1,3,5}
Padding	{None, Half of window size}
2nd pooling	
Type	{Max, Average}
Window size	[1,51]
Stride size	{1,3,5}
Padding	{None, Half of window size}
Global pooling	{None, Max pooling}
Output layer	{softmax, sigmoid}
Activation function	{ReLU, Leaky ReLU, Parametric ReLU}
Minibatch size	{32, 64, 128, 256, 512}
Batch normalization	{None, after conv.}
Dropout	{None, before output}
Optimizer	{Adam, AdaGrad}
Learning rate	{0.0001, 0.001, 0.01, 0.1}
Loss function	{Mean squared error, Cross entropy}

The output of the global pooling layer is a 64-dimensional vector that we named SCFP. We can construct a prediction model by using SCFP as input for fully connected layers. Specifically, we constructed a model that connected the SCFP and the output layers with one hidden layer. The model was trained using mini-batch stochastic gradient descent. Optimization was achieved by using Adam [22] with a learning rate of 0.01. All weights were initialized by a normal distribution with a mean of 0 and a standard deviation of 0.01. Other details are provided in Table 2.

We implemented our CNN using Python 3.5.2 and Chainer v1.24.0 [23].

### SMILES convolution fingerprint (SCFP)

Our CNN can be used not only as a prediction method but also as a method to compute a fingerprint. The 64-dimensional vector computed by the convolutional layers is a kind of fingerprint in the sense that it contains chemical structure information from a SMILES feature matrix (Fig. 2). In this regard, we designate this vector as the SMILES convolutional fingerprint (SCFP). Once the network is trained, we can compute SCFP for any compound not limited to those included in the training data. We propose to use SCFP as an alternative to conventional fingerprints such as ECFP. The advantage of SCFP over ECFP is that it can represent important features acquired from training. For example, if the network is trained for classifying the ligands of some protein, SCFP will represent the features that are important for discriminating the ligands from other compounds. This is in contrast to ECFP, which considers fixed types of features regardless of their application context. In the “Results” section, we demonstrate this nature of SCFP through its application to chemical space analysis.

### Chemical motif

**Fig. 3:**
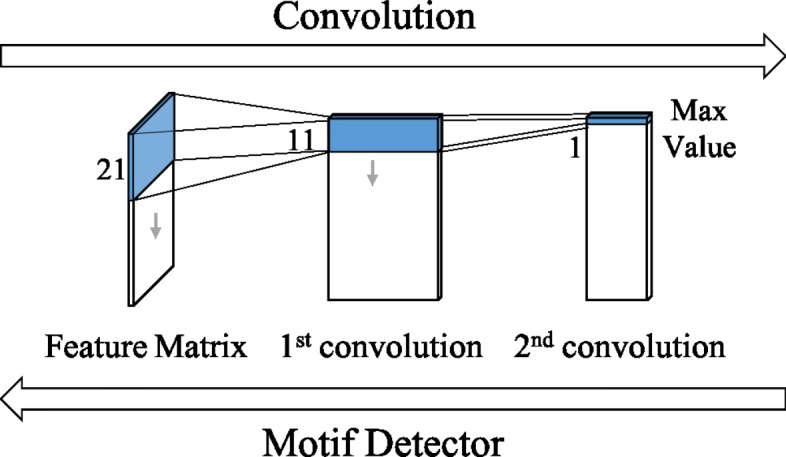
Detection of chemical motifs. Each dimension of SCFP is associated with the substructure of an input compound by tracing back through the CNN

Another merit of our CNN is its interpretability; i.e., it enables us to visualize the acquired features in SCFP as the substructures of an input compound. Since SCFP is computed by global max pooling, one dimension of SCFP corresponds to one of the filters in the second convolutional layer. As shown in Fig. 3, this allows us to associate each dimension with the substructure of an input compound by tracing back through the network. If a certain dimension takes a large value, it means a large contribution of the corresponding filter, thereby indicating the importance of the associated substructure. From this aspect, we designate such a substructure as the “chemical motif.” The analysis of chemical motifs facilitates the interpretation of prediction results by the network. For example, when we conduct ligand prediction, we can visualize and interpret chemical motifs as important substructures for ligand binding.

In practice, each dimension of SCFP may have a different value scale, making it difficult to compare across dimensions for identifying large-contribution filters. Thus, we normalize SCFP by the following procedure. First, we compute SCFP for all compounds in a given dataset. Then, we look at the values in the global max-pooling layer, and calculate their mean and variance for each filter over all compounds. Finally, we transform SCFP into Z-scores for each dimension by using the mean and the variance of the corresponding filter. For detecting chemical motifs, we focus on those dimensions of SCFP with Z-scores larger than 2.58 (i.e., 99% percentile). Note that this normalization procedure is only used for detecting chemical motifs, but not for training and prediction.

### Dataset and performance evaluation

In this study, we used the TOX 21 dataset [24] to evaluate the performance of our CNN. The TOX 21 dataset was originally created for the TOX 21 Challenge 2014, a competition of machine-learning methods for compound classification problems, and it has commonly been used as a benchmark dataset in many previous studies. The dataset contains information about whether approximately 8000 compounds would bind to 12 proteins. Tables 3 and 4 summarize the dataset. It consists of 12 subdatasets, each of which contains “active” and “inactive” compounds obtained from a specific experimental assay, and is divided into three types of data: “Train”, “Test”, and “Score”. The “Train” data are intended to be used as training data for machine-learning models. The “Test” data are intended to be used for the validation of models (e.g., hyperparameter optimization). The “Score” data are intended to be used for the final evaluation of model performance. Note that this nomenclature is not consistent with the standard terminology in machine learning: “Train”, “Test”, and “Score” data correspond to training, validation, and test data, respectively, in standard machine-learning terminology.

**Table 3:** TOX 21 assays

Subdataset	qHTS assay target
NR-AR	Androgen receptor using the MDA cell line
NR-AR-LBD	Androgen receptor ligand binding domain
NR-ER	Estrogen receptor *α* using the BG1 cell line
NR-ER-LBD	Estrogen receptor *α* ligand binding domain
NR-AhR	Aryl hydrocarbon receptor
NR-Aromatase	Aromatase enzyme
NR-PPAR- *γ*	Peroxisome proliferator-activated receptor *γ*
SR-ARE	Antioxidant response element
SR-ATAD5	Luciferase-tagged ATAD5 in human embryonic kidney cells
SR-HSE	Heat shock response
SR-MMP	Mitochondrial membrane potential
SR-p53	p53 response

**Table 4:** TOX 21 dataset

Subdataset	Train		Test		Score	
	Active	Inactive	Active	Inactive	Active	Inactive
NR-AR	380	8982	3	289	12	574
NR-AR-LBD	303	8296	4	249	8	574
NR-ER	937	6760	27	238	51	465
NR-ER-LBD	446	8307	10	277	20	580
NR-AhR	950	7219	31	241	73	537
NR-Aromatase	360	6866	18	196	39	489
NR-PPAR- *γ*	222	7962	15	252	31	574
SR-ARE	1098	6069	48	186	93	462
SR-ATAD5	338	8753	25	247	38	584
SR-HSE	248	7722	10	257	22	588
SR-MMP	1142	6178	38	200	60	483
SR-p53	537	8097	28	241	41	575

We evaluated the performance of the model by using the area under the receiver operating characteristic curve (ROC-AUC), which is a commonly used measure for evaluating the performance of classifiers. The ROC-AUC takes a value from 0 to 1, where a higher value indicates a more accurate classification between active and inactive compounds.

## Results

### Cross validation

We first trained and evaluated our CNN by using five-fold cross validation, giving several statistics such as computation time, memory usage, and convergence speed. We combined the three types of data (“Train”, “Test”, and “Score”) in the TOX 21 dataset into a single dataset, and performed a five-fold cross validation for the combined dataset. We continued the training until 300 epochs while measuring the ROC-AUC for validation. On average, the training took about 36 sec per epoch with several gigabytes of memory, and the ROC-AUC was converged at around 20 epochs. The detailed statistics for each subdataset is shown in Table 5.

**Table 5:** Summary of training statistics

Subdataset	Time (s/epoch)	Memory (MiB)	Convergence (epoch)
NR-AR	121.7	6551	15
NR-AR-LBD	12.9	6459	19
NR-ER	36.0	2763	17
NR-ER-LBD	37.7	2309	25
NR-AhR	13.3	1475	33
NR-Aromatase	15.2	6317	20
NR-PPAR- *γ*	2.7	4413	23
SR-ARE	16.7	1615	18
SR-ATAD5	74.7	4581	21
SR-HSE	49.0	3047	15
SR-MMP	40.3	3427	9
SR-p53	8.3	1211	11

The computation time is measured with a GPU server with NVIDIA Tesla P100 SXM2 16GB

**Fig. 4:**
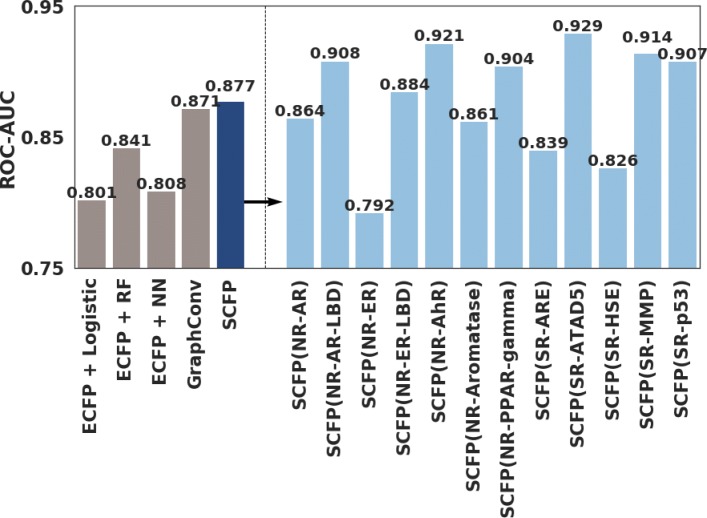
ROC-AUC of our model compared with those reported by previous studies. (Left) ROC-AUC averaged for 12 subdatasets were compared between our model (blue) and previous studies (gray). (Right) ROC-AUC of our model for each subdataset

We compared the ROC-AUC obtained by our model with conventional methods for compound classification problems. Specifically, the employed methods were: the logistic regression using ECFP as input, the random forest using ECFP as input, the deep neural network using ECFP as input [25], and the graph convolution proposed in [11]. The performance of our model was better than these existing methods (Fig. 4).

### Comparison with the winning model of TOX 21 challenge 2014

Next, we studied the potential of our CNN as a classification method by comparing its accuracy to that of the winning model of the TOX 21 Challenge 2014 [24]. For this purpose, we constructed a model where a fully-connected hidden layer is used between the SCFP and the output layers. We optimized the number of hidden units as well as the number and the size of filters in the first and the second convolution layers by using Bayesian optimization, GpyOpt [21].

We evaluated the performance of our model based on the same procedure as in the TOX 21 Challenge 2014. Specifically, we used the “Train” and the “Test” data to determine the hyperparameters, then evaluated the ROC-AUC using the “Score” data.

We compared our model to DeepTox [6], the winner method of TOX 21 Challenge 2014. The DeepTox authors used five variations of their model as follows: deep neural network (DNN) using only ECFP, DNN using ECFP and “DeepTox features” (proposed by the DeepTox authors), support vector machine (SVM) using ECFP and DeepTox features, random forest (RF) using ECFP and DeepTox features, and elastic net (ElNet) using ECFP and DeepTox features. In the DeepTox DNN model, the activation function of the hidden layers is ReLU, the sigmoid function is used for the final output, the mini-batch size is 512, and L2 regularization and dropout are used to prevent overfitting. DeepTox uses thousands of features consisting of 2500 in-house toxicophores features which comprise substructures previously reported as toxicophores, 200 in-house scaffold features that include the most common scaffolds that appear in organic molecules, and other 18 sets of features (the supplementary material of [6]).

Table 6 shows the results of the comparison of these models. On average, the ROC-AUC of our model was better than DNN using only ECFP, but slightly lower than those of the models using ECFP and “DeepTox features” except ElNet.

**Table 6:** Comparison of our CNN and DeepTox (the winning model of the TOX 21 Challenge 2014)

Input	Model	Ave.	AR	AR-LBD	ER	ER-LBD	AhR	Aromatase	PPAR- *γ*	ARE	ATAD5	HSE	MMP	p53
SMILES Matrix	CNN	0.813	0.789	0.793	0.776	0.765	0.905	0.786	0.791	0.754	0.803	0.835	0.928	0.832
ECFP	DNN	0.768	0.850	0.690	0.840	0.760	0.660	0.720	0.700	0.730	0.860	0.810	0.820	0.780
ECFP+DeepTox	DNN	0.837	0.778	0.825	0.791	0.811	0.923	0.804	0.856	0.829	0.775	0.863	0.930	0.860
ECFP+DeepTox	SVM	0.832	0.882	0.748	0.799	0.798	0.919	0.819	0.856	0.818	0.781	0.848	0.946	0.854
ECFP+DeepTox	RF	0.820	0.776	0.812	0.770	0.746	0.917	0.806	0.827	0.810	0.786	0.826	0.945	0.835
ECFP+DeepTox	ElNet	0.803	0.788	0.692	0.765	0.805	0.897	0.763	0.805	0.778	0.768	0.844	0.924	0.818

Our CNN takes SMILES feature matrices as input, while DeepTox uses ECFP and its original features

### Chemical space analysis with SCFP

**Fig. 5:**
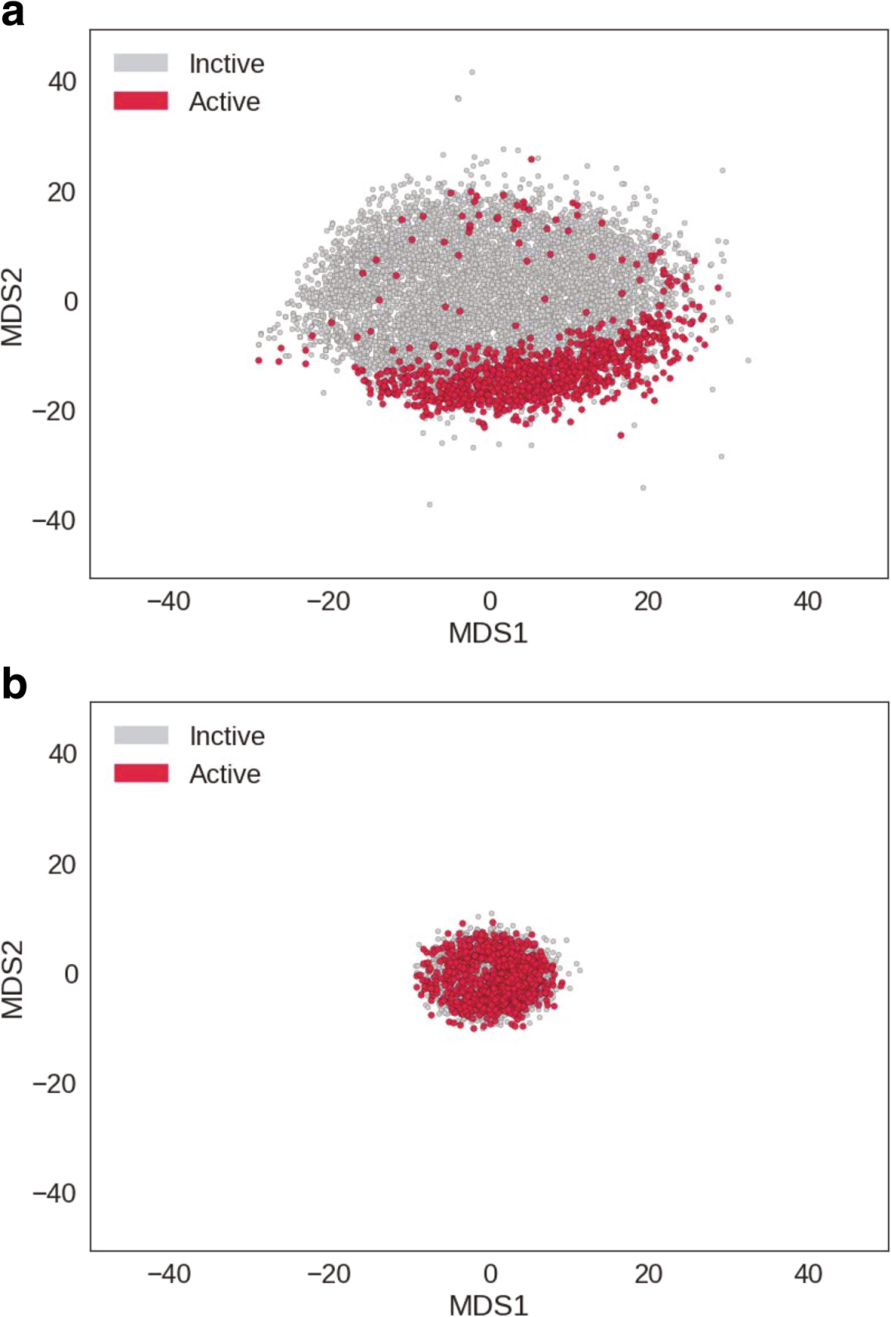
Chemical space analysis of the SR-MMP subdataset. SCFP (**a**) and ECFP (**b**) computed for all compounds in the dataset were plotted by MDS

To demonstrate that SCFP can be used as an alternative to conventional fingerprints, we conducted a chemical space analysis using SCFP. Specifically, we computed the SCFP for all compounds in the SR-MMP subdataset, and performed dimension reduction with multi-dimensional scaling (MDS). We also conducted a similar analysis using ECFP (length =1024, radius =2). Figure 5 compares the produced chemical space between SCFP and ECFP. In the chemical space produced by SCFP, active and inactive compounds were discriminated clearly. In contrast, ECFP failed to discriminate between the two groups in the chemical space. These results suggest that the expressive power of SCFP is stronger than that of ECFP for the chemical space analysis of the SR-MMP subdatasets.

Our results are especially surprising given the fact that the number of dimensions of SCFP (64) is much smaller than that of ECFP (1024). Although ECFP is often represented as a high-dimensional vector, the distance between fingerprints is not always proportional to the similarity of compounds because of hash collision. On the other hand, each element of SCFP represents the contribution of the corresponding substructure acquired from training. This means that the model preferentially extracts the substructure that greatly contributes to the label classification problem.

### Detection of chemical motifs

**Fig. 6:**
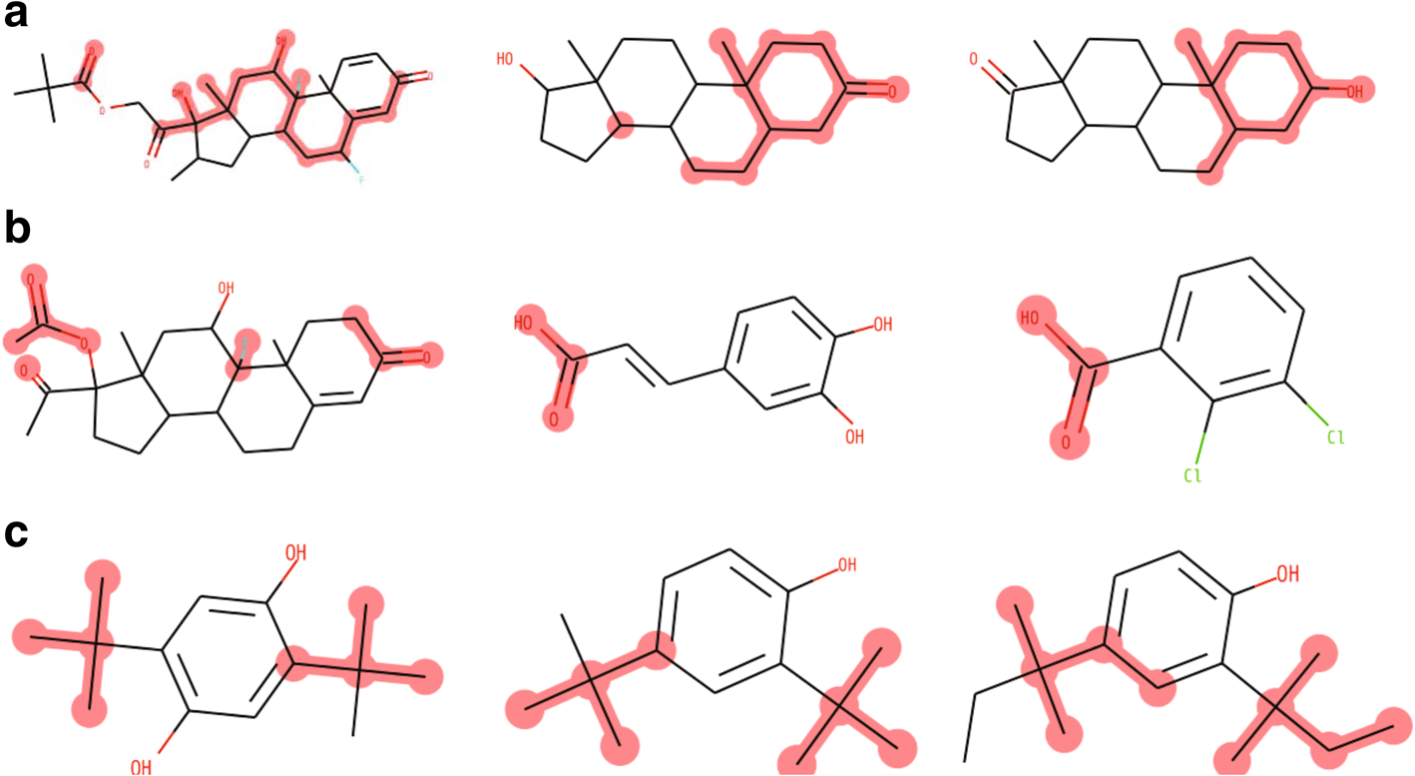
Examples of learned filters and chemical motifs for the NR-AR subdataset. **a** Filter 61 and corresponding chemical motifs on different compounds. **b** Filter 0 and corresponding chemical motifs on different compounds. **c** Filter 2 and corresponding chemical motifs on different compounds

Even though the prediction accuracy of our CNN was not substantially superior to that of the state-of-the-art method, our method has the advantage that it enables us to extract learned feature representation in the form of a chemical motif. Here, we present the analysis of chemical motifs using the NR-AR subdataset. We applied active compounds to our CNN, and detected chemical motifs as described in the “Methods” section. Figure 6 shows examples of the detected chemical motifs. These examples show that each filter corresponds to a distinct chemical motif in the compounds. Specifically, the filters 61, 0, and 2 represent, respectively, a steroid-like substructure (Fig. 6a), a substructure similar to a carboxy group (Fig. 6b), and a substructure similar to a *tert*-butyl group (Fig. 6c). By using this motif analysis, we can interpret these chemical motifs as important substructures for the NR-AR dataset, i.e., the binding of compounds to the androgen receptor (Table 3). Indeed, the steroid skeleton has been known as an important structure for the binding of the androgen receptor.

## Discussion

In this paper, we proposed a new CNN for analyzing chemical compound data. The CNN uses a SMILES-based feature matrix in a similar way to conventional CNNs for image data. We also developed a novel method for extracting acquired feature representation from our CNN as a form of chemical motif. Furthermore, we demonstrated that the analysis of chemical motifs greatly facilitates the interpretation of prediction results, highlighting the important substructures in a compound.

When used as a classification model, our CNN achieved higher accuracy than existing methods in the five-fold cross validation experiment (Fig. 4). In the TOX 21 Challenge 2014 experiment, our model was more accurate than DNN using only ECFP, but slightly less accurate than the models using ECFP and DeepTox features (Table 6). These “DeepTox features” significantly contributed to improving the accuracy of classification models. In this sense, SCFP automatically acquired by representation learning outperformed the previously well-used ECFP, but has not yet reached the performance of handcrafted DeepTox features especially tailored to TOX 21 Challenge.

SMILES feature matrices contain the structural properties of each atom (e.g., valence) in addition to the one-hot vector representing the atom symbol (Table 1). Although the one-hot vector has been commonly used as features to represent symbols in string data in machine learning, we did not simply follow such a strategy in this study. This is because the property of an atom changes substantially depending on its structural environment in a compound. For example, the property of a carbon atom is different depending on whether it is in a benzene ring, or is bonded to an oxygen atom. On the other hand, different kinds of atoms may have a similar property if they belong to the same family (i.e., group of elements in the periodic table), and their structural environments are similar. SMILES feature matrices were designed to capture this behavior by using the structural properties of atoms.

The merit of SMILES convolution is that it is unnecessary to specify substructures in advance as input features. Even when there is no prior knowledge about important substructures, our CNN can automatically acquire chemical motifs by representation learning. Moreover, since our CNN obtains important substructures preferentially, the size of the SCFP can be kept small (i.e., 64 in this study). This is in contrast to ECFP, which requires large-sized vectors for considering all possible substructures, but has limited expressive power due to hash collision.

In the analysis of chemical motifs, our CNN successfully detected a steroid-like chemical motif that has been known as an important structure for the binding of androgen receptors (Fig. 6a). The other detected motifs can be considered as candidates for novel skeleton structures for androgen receptors. Therefore, our proposed method has potential not only as a classification method, but also as a means of providing clues for drug discovery.

**Fig. 7:**
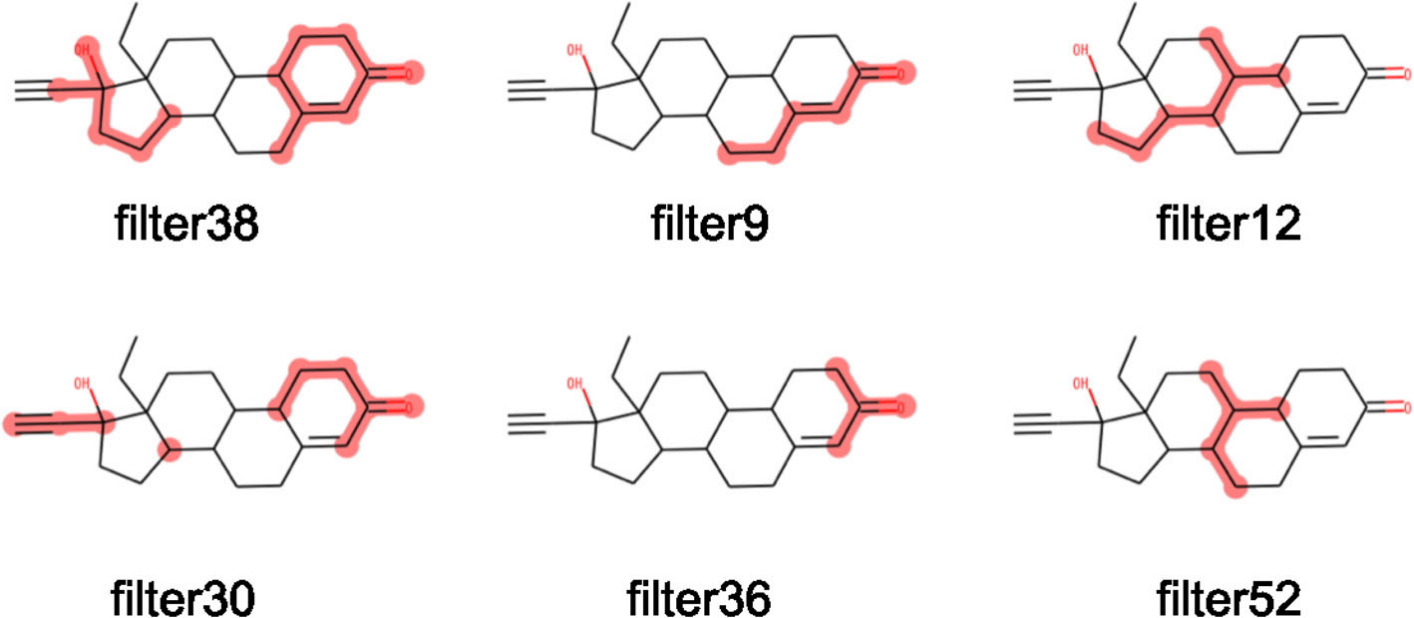
Filters representing similar chemical motifs. Each filter represents a similar but slightly different chemical motif

Since the detection of chemical motifs is based on filters, the size of the detectable chemical motifs is limited by the window size of filters. Specifically, the maximum motif size is 2*k*_1_+*k*_2_, where *k*_1_ and *k*_2_ are the window sizes in the first and second convolutional layers, respectively (i.e., 21 in this study; Fig. 3). However, as observed in Fig. 7, multiple filters may represent slightly distinct overlapping substructures and their combination may represent an entire motif. Thus, we expect the detection of large chemical motifs to be possible by the combined analysis of these filters.

The TOX 21 dataset is highly imbalanced between the number of active compounds and the number of inactive compounds. We attempted the following methods to resolve this imbalance.

- The learning rate was multiplied by a constant only for the positive data so that the positive data could be learned strongly.
- Only for active compounds, a compound was described also in non-canonical SMILES so that the number of positive examples was increased.

However, both methods did not contribute to improve the accuracy.

## Conclusions

In this study, we designed a feature matrix based on SMILES linear notation of compounds and applied it to our CNN where the convolution operation was performed only in one direction along the SMILES string. The performance of our CNN based on SMILES string was superior to that of the conventional fingerprint method used for the virtual screening of chemical compounds. In addition, the use of motif detection with learned filters not only enabled important known substructures such as protein-binding sites but also substructures of unknown functional groups to be detected. Using the TOX 21 Challenge as benchmark, we achieved performance comparable to that of the current winning model. Furthermore, multivariate analysis confirmed that the chemical space consisting of the features learned by SMILES-based representation learning were able to adequately express a rich feature space that enabled the accurate discrimination of compounds.

## Abbreviations

CNN: Convolutional neural network
DNN: Deep neural network
ECFP: Extended-connectivity fingerprint
ElNet: Elastic net
MDS: Multi-dimensional scaling
RF: Random forest
ROC-AUC: The area under the receiver operating characteristic curve
SCFP: SMILES convolution fingerprint
SVM: Support vector machine
